# Evaluation of optimum classification measures used to define textbook outcome among patients undergoing curative-intent resection of gastric cancer

**DOI:** 10.1186/s12885-023-11695-4

**Published:** 2023-12-06

**Authors:** L Bobrzynski, K Sędłak, K Rawicz-Pruszyński, P Kolodziejczyk, A Szczepanik, W Polkowski, P Richter, M Sierzega

**Affiliations:** 1 First Department of Surgery, Jagiellonian University Medical College, 2 Jakubowskiego Street, Krakow, 30-688 Poland; 2 Department of Surgical Oncology, Medical University of Lublin, Lublin, Poland

**Keywords:** Gastric cancer, Textbook outcome, Prognosis

## Abstract

**Background:**

Textbook outcome (TO) is a composite measure reflecting various aspects of services provided to patients with solid malignancies. We sought to evaluate the importance of various TO components previously proposed for gastric cancer.

**Methods:**

Prospectively maintained electronic databases of 1,743 patients treated in two academic surgical centres were reviewed. Six candidate definitions of TO were evaluated based on their ability to accurately predict patients’ prognosis by Cox proportional hazards modelling.

**Results:**

TO definition combining 10 measures corresponding to complete tumour resection with an uneventful postoperative course showed the best goodness of fit by achieving the lowest values of Akaike (AIC) and Bayesian (BIC) information criteria and the best predictive performance based on the highest value of c-index. The overall median survival was significantly longer for patients with than without textbook outcome (69.0 *vs* 20.1 months, *P* < 0.001). TO maintained its prognostic value in a multivariate model controlling for age, sex, comorbidities, treatment, and tumour related variables and was associated with a 39% lower risk of death (HR 0.61, 95%CI 0.51 – 0.73, *P* < 0.001). Nine variables identified as predictors of TO were used to develop a nomogram showing very good correlation between the predicted and actual probability of achieving TO. The AUC of ROC obtained from the nomogram was 0.752 (95% CI 0.727 to 0.781).

**Conclusions:**

A uniform definition of textbook outcome provides clinically relevant prognostic information and could be used in quality improvement programs for gastric cancer patients.

**Supplementary Information:**

The online version contains supplementary material available at 10.1186/s12885-023-11695-4.

## Introduction

Gastric cancer (GC) remains the fifth most frequently diagnosed malignancy and the third leading cause of cancer death [1]. However, despite various attempts to harmonise clinical pathways, there is substantial heterogeneity in surgical and oncological services provided to patients [2–4]. Furthermore, several population databases demonstrated that some aspects of care for GC patients are suboptimal, including inadequate lymph node dissection or unexpectedly high morbidity and mortality rates [5, 6].

The need for better quality indicators applicable to complex operative procedures started an increasing interest in composite measures of surgical performance [7, 8]. A combination of several well-established parameters, usually including rates of mortality, morbidity, readmissions, or length of hospital stay, has been proposed as a more accurate approximation for the complexity of the surgical care than any individual parameter [9, 10]. This is particularly important for oncological surgery, where quality refers not only to short term outcomes but also long-term survival. Textbook outcome (TO), incorporating several anticipated postoperative endpoints across all important domains of surgical performance, represents an ideal (so-called textbook) perioperative course. Its suitability to evaluate various aspects of surgical quality has been demonstrated for both oncological [11–14] and general surgery [15–17]. TO has also been investigated in few population-based cohort studies recruiting patients with gastric cancer [18–20]. The data obtained corroborated the anticipated variability in quality of care and identified the limiting factors for achieving TO. Moreover, they suggested significant correlation between accomplishing TO and superior long-term survival.

Despite the overall optimism, there are still some important aspects related to the applicability of TO as a validated measure of clinical pathways for gastric cancer. Harmonisation is one of the unresolved issues as the previously proposed definitions showed marked variability across studies [18–23]. Therefore, the objective of the current study was to evaluate candidate components of TO and identify those most relevant for patients’ prognosis. Subsequently, we developed and validated a nomogram predicting TO after curative-intent resection of gastric cancer.

## Methods

### Patient population

Prospectively maintained electronic databases of patients with gastric adenocarcinoma undergoing gastrectomy with curative intent in two academic surgical centres were reviewed. The Kraków cohort included patients treated in the First Department of Surgery, Jagiellonian University Medical College between 1996 and 2020. The Lublin cohort recruited patients treated in the Department of Surgical Oncology, Medical University of Lublin between 2010 and 2020. The first cohort was used to evaluate the candidate definitions of TO and identify potential predicting factors. Subsequently, both cohorts were used to develop and validate a nomogram predicting the odds of achieving TO (Figure S1, supporting information). This study has been approved by the Bioethics Committee of the Jagiellonian University.

### Outcome measures

Data related to surgical treatment and staging were reclassified according to the most recent guidelines [24, 25]. The postoperative course was evaluated using the Clavien-Dindo classification of surgical complications [26]. TO was defined using six definitions previously reported for gastric cancer (Table S1, supporting information) [18–23]. Individual components used to formulate TO included macroscopically complete resection according to the surgeon, microscopically radical resection by the pathologist (R0), minimum number of lymph nodes retrieved and examined, no intraoperative complication, no grade II or higher postoperative complication according to the Clavien–Dindo classification, no reintervention (surgical, endoscopic or radiological), no readmission to the ICU, no postoperative mortality, duration of hospital stay, no readmission within 30 days after discharge from hospital, and compliance with chemotherapy as recommended by the National Comprehensive Cancer Network (NCCN) clinical practice guidelines. The minimum number of lymph nodes was evaluated adopting two cut-off values of 15 and 16 nodes [19, 22]. Prolonged hospitalization was defined as hospital stay exceeding 19 days, 21 days, and 75^th^ percentile of the cohort [19, 22, 23]. Postoperative mortality was defined as in-hospital death or death within 30 days or 90 days after surgery [19, 20]. The primary evaluation criterion for TO was overall survival defined as time from the date of surgical resection to the date of death from any cause or date of the last follow-up. All death dates were verified by data from the national census office.

### Statistical analysis

The differences in proportions between groups were evaluated using the chi-square test, and the Mann–Whitney U test was used to detect differences in quantitative variables. Overall and conditional survival (under the condition of surviving the first postoperative 30 days and hospital stay) was analysed according to the Kaplan–Meier method and the log-rank test was used to detect differences between groups. Multivariate analysis was performed using a Cox proportional hazards model with a backward stepwise selection procedure. The goodness of fit of individual Cox models were compared using the Akaike (AIC) and Bayesian information criterion (BIC), while their predictive value with Harrell's concordance index (c-index) [27, 28].

After checking for multicollinearity, multivariable logistic regression was used to identify variables associated with achieving a textbook outcome. Three penalized regression methods (ridge, lasso, and elastic net regression) were used for selection of predictive variables and formulation of a multivariable logistic regression model based on minimising the value of AIC [29]. Internal validation of the predictive model was performed by bootstrapping with 1000 resamples [30]. Calibration plots were generated to evaluate the accuracy of prediction and model discrimination was quantified using the c-index corresponding to the area under the Receiver Operating Characteristic (ROC) curve. A nomogram was formulated based on the results of the logistic regression model. The significance level (*P*) < 0.05 in a two-tailed test was considered statistically significant. All statistical analyses were performed using the IBM® SPSS® Statistics 28 software package (IBM Corporation, NY) and RStudio (Integrated Development Environment for R) version 2021.9.2.382 with packages survival (3.3–1), rms (6.2–0), pROC (1.16.2), and caret (6.0–91).

## Results

### Study population

The study population includes 1,743 patients with non-metastatic gastric adenocarcinoma treated in two academic surgical centres, corresponding to Kraków (*n* = 1,479) and Lublin (*n* = 264) cohorts. Baseline patient characteristics is shown in Table 1.

**Table 1:** Baseline characteristics of patients

**Characteristic**	**Cohort**			***P***
	Overall	Kraków	Lublin	
	(*N* = 1,743)	(*N* = 1,479)	(*N* = 264)	
Gender, male	1,177 (68%)	1,018 (69%)	159 (60%)	0.006
Age, years	65 (55, 72)	65 (56, 73)	61 (53, 69)	< 0.001
Tumour location				< 0.001
lower	606 (35%)	540 (37%)	66 (25%)	
middle	604 (35%)	485 (33%)	119 (45%)	
upper	420 (24%)	341 (23%)	79 (30%)	
diffuse infiltrative	113 (6.5%)	113 (7.6%)	0 (0%)	
Tumour size, mm	50 (30, 80)	52 (30, 80)	31 (20, 50)	< 0.001
Lauren type				< 0.001
intestinal	871 (50%)	742 (50%)	129 (49%)	
diffuse	632 (36%)	556 (38%)	76 (29%)	
mixed	240 (14%)	181 (12%)	59 (22%)	
Tumour grade				< 0.001
1	207 (12%)	196 (13%)	11 (4.2%)	
2	513 (30%)	400 (27%)	113 (43%)	
3	1,016 (59%)	877 (60%)	139 (53%)	
Type of gastrectomy				< 0.001
distal	484 (28%)	403 (27%)	81 (31%)	
proximal	181 (10%)	132 (8.9%)	49 (19%)	
total	1,078 (62%)	944 (64%)	134 (51%)	
Lymph nodes examined	26 (17, 37)	24 (15, 32)	28 (18, 38)	< 0.001
Multivisceral resection	507 (29%)	443 (30%)	64 (24%)	0.06
Neoadjuvant treatment	363 (21%)	156 (11%)	207 (78%)	< 0.001
pT classification				0.003
T1a	140 (8.0%)	112 (7.6%)	28 (11%)	
T1b	149 (8.5%)	132 (8.9%)	17 (6.4%)	
T2	194 (11%)	150 (10%)	44 (17%)	
T3	763 (44%)	651 (44%)	112 (42%)	
T4a	287 (16%)	244 (16%)	43 (16%)	
T4b	210 (12%)	190 (13%)	20 (7.6%)	
pN classification				< 0.001
N0	635 (36%)	513 (35%)	122 (46%)	
N1	226 (13%)	191 (13%)	35 (13%)	
N2	241 (14%)	199 (13%)	42 (16%)	
N3a	331 (19%)	297 (20%)	34 (13%)	
N3b	310 (18%)	279 (19%)	31 (12%)	

Data are expressed as median (interquartile range) or number (%); the Mann–Whitney U test was used to evaluate differences in quantitative variables and the differences in proportions were evaluated using the chi-square test

### Identification of optimum TO measures (Kraków cohort)

At the time of final follow-up, 1059 of 1479 (72%) patients in the Kraków cohort had died. The median follow-up for all surviving subjects was 101 months. Six Cox proportional hazards models were developed corresponding to previously published definitions of TO (Table S2, supporting information). Of all models tested, the model formulated by Busweiler et al. showed the best goodness of fit by achieving the lowest values of AIC and BIC and the best predictive performance based on the highest value of c-index. Therefore, the 10-item definition was selected as the optimum definition of TO for the current study (no intraoperative complication, macroscopically and microscopically radical resection, ≥ 15 lymph nodes examined, no Clavien–Dindo grade II or higher complication, no reintervention within 30 days after surgery, no readmission to ICU within 30 days after surgery, no postoperative mortality within 30 days after surgery, hospital stay ≤ 21 days, no hospital readmission within 30 days after discharge). Supplementary Figure S2 and S3 shows Kaplan–Meier overall and conditional survival curves for patients achieving individual TO metrics except the item referring to postoperative 30-day mortality. Apart from hospital readmission within 30 days and the number of evaluated lymph nodes, all individual parameters were associated with superior survival by the Cox proportional hazards model (Table S3, supporting information). Moreover, there was a gradual reduction in relative hazard of death when increasing the number of achieved TO measures from 5 or less items up to 10 items (Table S4, supporting information).

### Trends in TO (Kraków cohort)

**Fig. 1:**
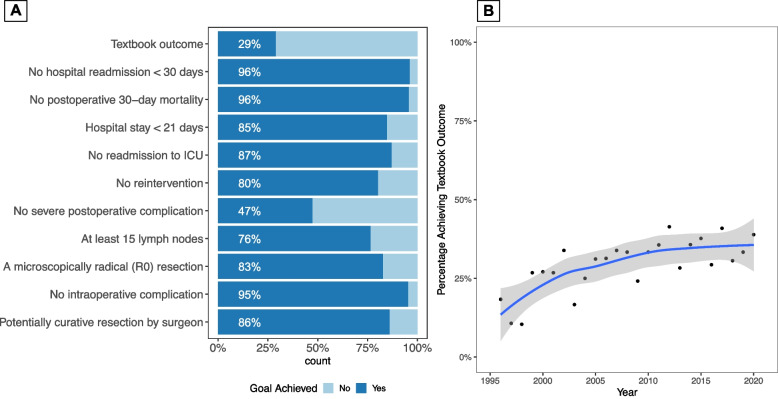
Textbook outcome (TO). **A** Proportion of patients achieving each quality metric of TO. **B** LOESS curve fitting (solid line with 95% confidence intervals) for the temporal trend in the annual proportion of patients achieving TO

The absence of Clavien-Dindo grade 2 or higher complications (47%) was the most common limiting factor for TO, while the prevalence of other factors did not exceed 24% (Fig. 1). LOESS curve fitting showed a gradual increase in the annual proportion of patients achieving TO from 18.4% in 1996–2000 to 34.3% in 2016–2020 (*P* = 0.001). This trend was largely due to increased rates of resections with curative intent, lack of intraoperative complications, resections with at least 15 lymph nodes, and shortened hospital stay (Figure S4, supporting information). No major variability was found for 30-day mortality, 30-day readmissions, and microscopically radical resections. Figure S5 demonstrates changes in Clavien-Dindo severity scores according to the study period and figure S6 shows severity scores related to the type of surgery.

### Prognostic implications of TO (Kraków cohort)

**Fig. 2:**
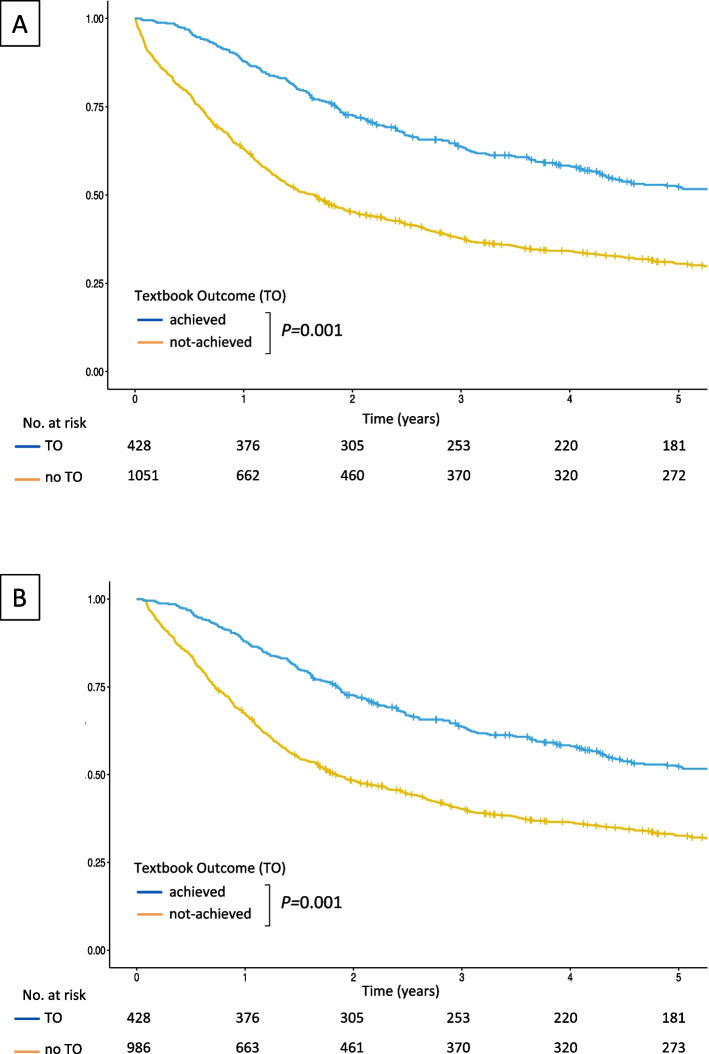
Kaplan–Meier survival curves for overall (**A**) and conditional (**B**) survival of patients who underwent surgery for gastric cancer for groups with and without a Textbook Outcome (TO) (log rank test)

The overall median survival was significantly longer for patients with (69.0 months, 95% CI 53.3–96.3) than without textbook outcome (20.1 months, 95% CI 17.0–22.4, *P* < 0.001) (Fig. 2). Since postoperative mortality is a component of TO, a conditional survival analysis was also carried out excluding deaths in-hospital and within 30 days after surgery, producing similar Kaplan–Meier survival curves. Additionally, TO maintained its prognostic value in a Cox proportional hazards model controlling for age, sex, comorbidities, treatment, and tumour related variables (Table S5, supporting information) and was associated with a 39% lower risk of death (HR 0.61, 95% CI 0.51 – 0.73, *P* < 0.001).

### Factors associated with TO (Kraków cohort)

**Fig. 3:**
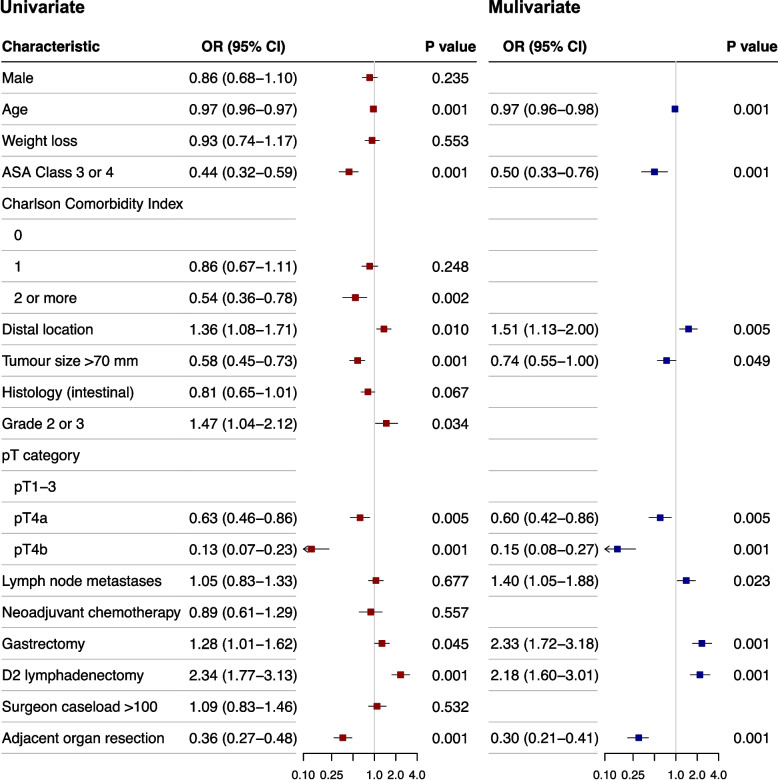
Factors associated with Textbook Outcome by univariable and multivariable logistic regression analysis. Odds ratio (OR) represents coefficients derived from the final model

Figure 3 shows odds ratios for all the variables potentially associated with TO by univariate analysis and the final model coefficients identified by multivariable regression. Patients’ age, ASA class 3 or 4, tumour size greater than 70 mm, tumour infiltrating gastric serosa or surrounding organs (pT4a or pT4b), and multivisceral resections were associated with a lower likelihood of achieving TO. Patients with lymph node metastases, tumours located in the distal third of the stomach, as well as treated by total gastrectomy or D2 lymphadenectomy had an increased odds of having TO.

### Nomogram predicting TO (Kraków and Lublin cohort)

**Fig. 4:**
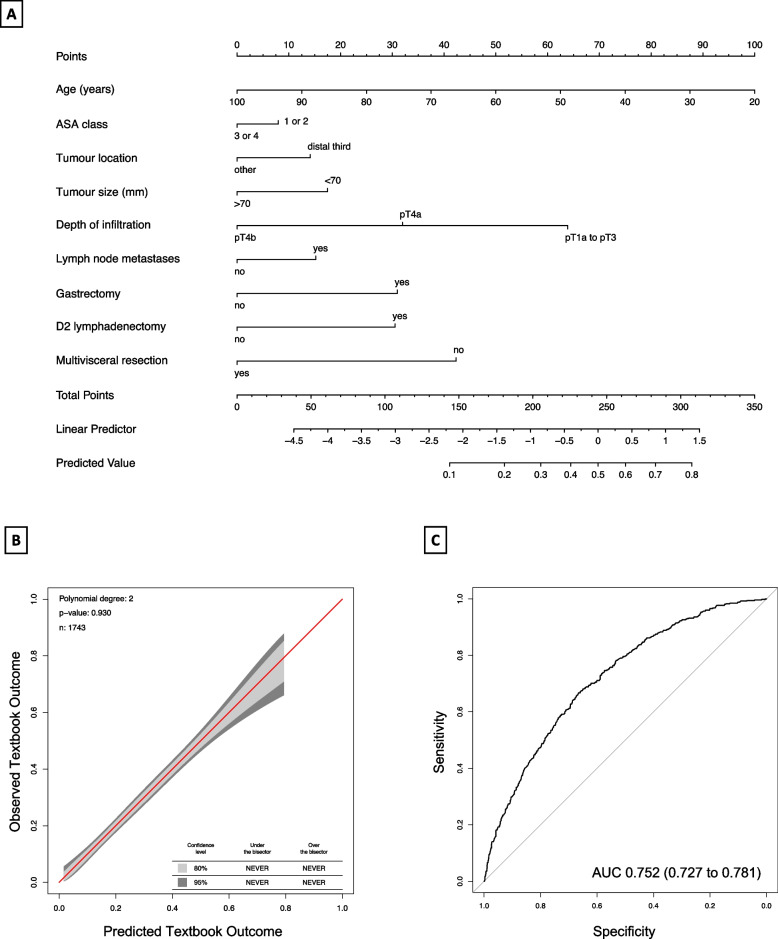
Nomogram predicting the likelihood of achieving Textbook Outcome (TO) after gastrectomy for gastric cancer. The nomogram is used by adding up the points identified on the points scale for each variable. The total points projected on the bottom scale indicate the probability of achieving TO. ASA – American Society of Anesthesiologists, G1 – well differentiated, G2/G3 – moderately/poorly differentiated

Nine variables identified as predictors of TO were used to develop a nomogram combining datasets from two patient cohorts. Figure 4 shows the graphical representation of the nomogram. Model calibration by bootstrapping and calibration belt showed very good correlation between the predicted and actual probability of achieving TO. The AUC of ROC obtained from the logistic regression model was 0.752 (95% CI 0.727 – 0.781).

## Discussion

Textbook outcome (TO), representing an ideal hospitalization, has been proposed as a clinically relevant measure reflecting the complexity of perioperative care for various types of cancers. In the present study, we have verified validity of the current definitions of TO among patients undergoing curative-intent resection of gastric cancer. Moreover, we identified factors associated with the likelihood of achieving TO and developed a nine-item nomogram for precisely estimating the probability of completing TO.

The idea of TO meets the needs of patients and healthcare systems to develop an easy to interpret measure of complex cancer care combining parameters quantifying safety and quality of surgery [31]. The proportion of patients completing the ideal hospitalization reported in previous studies on gastric cancer ranged from 22 to 51%, reaching in some Asian centres 72% [18–21, 32–36]. Postoperative complications and the low number of evaluated lymph nodes were the most common limiting factors, but also those responsible for gradually increasing trends in TO [18–20, 22, 33–37].

Though TO seems to be a potentially useful tool for communicating and comparing outcomes for complex oncologic procedures, there are challenges with the optimum construction of its elements. Previous reports regarding TO in abdominal surgery clearly suggested the existence of organ-specific or disease-specific factors associated with an ideal perioperative course. Its suitability to evaluate various aspects of surgical quality has been demonstrated for both oncological [11–14] and general surgery [15–17]. TO has also been investigated in few population-based cohort studies recruiting patients with gastric cancer [18–20].

Initial components of TO for patients with gastric cancer were defined by expert opinion and included 10 measures related to safety (intraoperative and postoperative complications, reinterventions, mortality, ICU and hospital stay, readmission after discharge) and efficacy of treatment (resection margins, number of evaluated lymph nodes) [19]. The criteria proposed by Busweiler et al. were subsequently used for oesophago-gastric surgery in other studies [11, 32–35]. However, another definitions of TO or textbook oncologic outcome (TOO) were published including four [22, 23], eight [18, 20] or nine [21] components. Moreover, the alterations included not only the number of individual indicators, but also their definitions like the minimum number of lymph nodes (15 [19] or 16 [22]), prolonged hospital stay (19 days, [23] 21 days, [19] or 75^th^ percentile of the cohort [22]), and postoperative mortality (30 days [19] or 90 days [20]). Consequently, the prevalence of TO among patients undergoing surgery for gastric cancer is markedly affected by the number of measures used to define this outcome [38, 39].

To our knowledge, this is the first study to assess the optimum definition of TO for gastric cancer that was subsequently used to develop and validate a nomogram tool predicting an ideal perioperative course. Using prospectively collected datasets we had the unique opportunity to evaluate all components of TO proposed in the literature, avoiding the risks of incomplete data or lack of standard definitions as previously encountered by some population-based registries [18, 20, 37]. In order to evaluate the generalizability of results, the datasets covered broad time periods, potentially reflecting the evolving standards for patient care. A detailed comparison of six potential definitions found the 10-item TO proposed by Busweiler et al. as the most informative and most precisely reflecting the likelihood to achieve long-term survival. Moreover, there was a correlation between the number of achieved individual measures and patients’ prognosis. Therefore, the selected definition seems to be most appropriate for further studies evaluating clinical pathways for gastric cancer, even though it requires access to some data not routinely collected by administrative databases.

Essentially, all previous studies demonstrated impaired survival among patients with gastric cancer who failed to achieve TO [18, 20, 22, 23, 32, 33, 35]. A Netherland national cohort study of 2,769 patients included in the DUCA registry between 2011 and 2016 reported significantly reduced hazard ratio (HR) of death associated with TO (10 measures) for both overall survival (HR 0.62, 95% CI 0.54 to 0.71) and conditional survival (HR 0.69, 95% CI 0.60 to 0.79) [32]. Another cohort study recorded data of 1,836 patients from the Population Registry of Esophageal and Stomach Tumours in Ontario (PRESTO) between 2004 and 2015 [18]. They found a 41% decrease in the relative risk of death (HR 0.59, 95% CI 0.48 to 0.72) among patients achieving TO (8 measures). Similar findings were reported using data for 1,293 patients from the population-based Spanish EURECCA Registry recorded between 2014 and 2017. Using Cox regression modelling, the authors showed that TO (8 measures) caused a 33% reduction in the relative risk of death (HR 0.67, 95%CI 0.55 to 0.83) [20]. Altogether, data from these three population-based registries and the current study demonstrated a clear correlation between TO and patients’ survival. The underlying mechanism for such an association is most likely multidimensional, as TO combines several factors that could influence prognosis, including resection margins, precise evaluation of lymph nodes, and postoperative complications.

Given the prognostic implications of TO, identification of factors associated with the likelihood of achieving the desired outcome could provide clinically relevant benefits. In the original study of Busweiler et al., ASA grade ≥ 3 (OR 0.74), Charlson co-morbidity index score ≥ 2 (OR 0.74), clinical tumour stage III (OR 0.61), no neoadjuvant therapy (OR 0.75), and resection of additional organs (OR 0.66) significantly decreased the likelihood of textbook outcome [19]. Data from the Canadian PRESTO registry identified younger age, fewer concomitant disorders, neoadjuvant chemotherapy, distally located tumours, and lower T stage as factors increasing the odds for TO [18]. In a Spanish population-based analysis, age > 64 years, Charlson comorbidity index ≥ 3, neoadjuvant chemoradiotherapy, multivisceral resection, and surgery performed in a community hospital were associated with the lower odds of achieving TO [20]. Although data from these three population-based studies suggested the ability to predict TO using relatively simple criteria, no validated tools were available so far. Therefore, we aimed to develop and validate a nomogram allowing accurate prediction of TO. First, we screened potential variables associated with the likelihood of TO using a single centre dataset. Subsequently, we developed a 9-item nomogram using two independent and heterogenous datasets covering broad time periods with different standards for patient care, including perioperative treatment. The prepared nomogram showed acceptable performance, suggesting potential applicability for further clinical endorsement.

Our results provide a clinically relevant rationale for the use of carefully selected textbook outcome measures as a source of prognostic information. However, some important limitations of the current study should be considered. First, a retrospective analysis of prospectively collected data cannot eliminate the risk of selection bias. Second, we used overall survival as the primary criterion to evaluate TO. Although a similar approach was used by previous studies, adoption of cancer-specific survival could provide some additional insight. Third, the overall proportion of patients given preoperative chemotherapy was 21%, and this was relatively low compared to the current guidelines. However, one of the aims of our study was to develop and evaluate a nomogram predicting TO applicable for different clinical situations, including various therapeutic regimens. Consequently, we were able to demonstrate the validity of the nomogram in two different populations with a low and high prevalence of neoadjuvant treatment.

In summary, this study identified the optimum component measures of Textbook Outcome associated with long-term survival of patients undergoing curative-intent resection of gastric cancer. Additionally, we developed a nomogram applicable for predicting the likelihood of achieving TO and validated it in multicenter settings. Further research is needed to assess if such tools could be used in quality improvement programs for gastric cancer patients.

### Supplementary Information

**Additional file 1:** **Table S1.** Components used to define Textbook Outcome (TO) in gastric cancer surgery. **Table S2.** Performance parameters of Cox proportional hazards models for Textbook Outcomes (TO) defined using various components (Kraków cohort, *N =* 1,479). **Table S3.** Cox proportional analysis for overall and conditional survival in patients with individual components of textbook outcome (Kraków cohort, *N =* 1,479). **Table S4.** Univariate analysis of overall and conditional survival in patients with individual components of textbook outcome (TO) (Kraków cohort, *N =* 1,479). Table S5. Univariate and multivariate Cox proportional hazards analysis for overall survival (Kraków cohort, *N =* 1,479). **Figure S1.** Study flowchart. **Figure S2.** Kaplan–Meier survival curves for overall survival of patients achieving each quality metric of Textbook Outcome for Kraków cohort, *N =* 1,479 (log-rank test). **Figure S3.** Kaplan–Meier survival curves for conditional survival of patients achieving each quality metric of Textbook Outcome for Kraków cohort, *N =* 1,479 (log-rank test). **Figure S4.** LOESS curve fitting (solid line with 95% confidence intervals) for the temporal trend in the annual proportion of patients achieving each quality metric of Textbook Outcome (Kraków cohort, *N =* 1,479). **Figure S5.** Distribution of postoperative complications according to the Clavien-Dindo classification in different time periods (Kraków cohort, *N =* 1,479). **Figure S6.** Distribution of postoperative complications according to the Clavien-Dindo classification by the type of resection (Kraków cohort, *N =* 1,479).

## Data Availability

The datasets generated during and/or analysed during the current study are available from the corresponding author on reasonable request.

## References

[1] Bray F, Ferlay J, Soerjomataram I, Siegel RL, Torre LA, Jemal A (2018). Global cancer statistics 2018: GLOBOCAN estimates of incidence and mortality worldwide for 36 cancers in 185 countries. CA Cancer J Clin.

[2] Dikken JL, van Sandick JW, Allum WH, Johansson J, Jensen LS, Putter H (2013). Differences in outcomes of oesophageal and gastric cancer surgery across Europe. Br J Surg.

[3] Messager M, de Steur WO, van Sandick JW, Reynolds J, Pera M, Mariette C (2016). Variations among 5 European countries for curative treatment of resectable oesophageal and gastric cancer: A survey from the EURECCA Upper GI Group (EUropean REgistration of Cancer CAre). Eur J Surg Oncol.

[4] Yamamoto M, Rashid OM, Wong J (2015). Surgical management of gastric cancer: the East vs West perspective. J Gastrointest Oncol.

[5] Datta J, Lewis RS, Mamtani R, Stripp D, Kelz RR, Drebin JA (2014). Implications of inadequate lymph node staging in resectable gastric cancer: a contemporary analysis using the National Cancer Data Base. Cancer.

[6] Baiocchi GL, Giacopuzzi S, Reim D, Piessen G, Costa PMD, Reynolds JV (2020). Incidence and Grading of Complications After Gastrectomy for Cancer Using the GASTRODATA Registry: A European Retrospective Observational Study. Ann Surg.

[7] Yu J, Massarweh NN (2019). Surgical Quality Improvement: Working Toward Value or a Work in Progress?. J Surg Res.

[8] Nolan T, Berwick DM (2006). All-or-none measurement raises the bar on performance. JAMA.

[9] Mehta R, Tsilimigras DI, Paredes A, Dillhoff M, Cloyd JM, Ejaz A (2021). Assessment of hospital quality and safety standards among Medicare beneficiaries undergoing surgery for cancer. Surgery.

[10] Dimick JB, Staiger DO, Osborne NH, Nicholas LH, Birkmeyer JD (2012). Composite measures for rating hospital quality with major surgery. Health Serv Res.

[11] Kalff MC, Vesseur I, Eshuis WJ, Heineman DJ, Daams F, van der Peet DL (2021). The Association of Textbook Outcome and Long-Term Survival After Esophagectomy for Esophageal Cancer. Ann Thorac Surg.

[12] Kulshrestha S, Bunn C, Patel PM, Sweigert PJ, Eguia E, Pawlik TM (2020). Textbook oncologic outcome is associated with increased overall survival after esophagectomy. Surgery.

[13] Merath K, Chen Q, Bagante F, Alexandrescu S, Marques HP, Aldrighetti L (2019). A Multi-institutional International Analysis of Textbook Outcomes Among Patients Undergoing Curative-Intent Resection of Intrahepatic Cholangiocarcinoma. JAMA Surg.

[14] van Roessel S, Mackay TM, van Dieren S, van der Schelling GP, Nieuwenhuijs VB, Bosscha K (2020). Textbook Outcome: Nationwide Analysis of a Novel Quality Measure in Pancreatic Surgery. Ann Surg.

[15] Halpern SE, Moris D, Shaw BI, Kesseli SJ, Samoylova ML, Manook M (2021). Definition and Analysis of Textbook Outcome: A Novel Quality Measure in Kidney Transplantation. World J Surg.

[16] Kuhrij LS, Karthaus EG, Vahl AC, Willems MCM, Elshof JW, de Borst GJ (2020). A Composite Measure for Quality of Care in Patients with Symptomatic Carotid Stenosis Using Textbook Outcome. Eur J Vasc Endovasc Surg.

[17] Poelemeijer YQM, de Marang-vanMheen PJ, Wouters M, Nienhuijs SW, Liem RSL (2019). Textbook Outcome: an Ordered Composite Measure for Quality of Bariatric Surgery. Obes Surg.

[18] Levy J, Gupta V, Amirazodi E, Allen-Ayodabo C, Jivraj N, Jeong Y (2022). Textbook Outcome and Survival in Patients With Gastric Cancer: An Analysis of the Population Registry of Esophageal and Stomach Tumours in Ontario (PRESTO). Ann Surg.

[19] Busweiler LA, Schouwenburg MG, van Berge Henegouwen MI, Kolfschoten NE, de Jong PC, Rozema T (2017). Textbook outcome as a composite measure in oesophagogastric cancer surgery. Br J Surg.

[20] Dal Cero M, Roman M, Grande L, Yarnoz C, Estremiana F, Gantxegi A (2022). Textbook outcome and survival after gastric cancer resection with curative intent: A population-based analysis. Eur J Surg Oncol.

[21] Roh CK, Lee S, Son SY, Hur H, Han SU (2021). Textbook outcome and survival of robotic versus laparoscopic total gastrectomy for gastric cancer: a propensity score matched cohort study. Sci Rep.

[22] Spolverato G, Paro A, Capelli G, Dalmacy D, Poultsides GA, Fields RC (2022). Surgical treatment of gastric adenocarcinoma: Are we achieving textbook oncologic outcomes for our patients?. J Surg Oncol.

[23] Aquina CT, Hamad A, Becerra AZ, Cloyd JM, Tsung A, Pawlik TM (2021). Is Textbook Oncologic Outcome a Valid Hospital-Quality Metric after High-Risk Surgical Oncology Procedures?. Ann Surg Oncol.

[24] Japanese Gastric Cancer A (2021). Japanese gastric cancer treatment. Japanese gastric cancer treatment guidelines 2018 (5th edition).

[25] Brierley JD, Gospodarowicz MK, Wittekind C (2017). TNM Classification of Malignant Tumours.

[26] Dindo D, Demartines N, Clavien PA (2004). Classification of surgical complications: a new proposal with evaluation in a cohort of 6336 patients and results of a survey. Ann Surg.

[27] McLernon DJ, Giardiello D, Van Calster B, Wynants L, van Geloven N, van Smeden M (2023). Assessing Performance and Clinical Usefulness in Prediction Models With Survival Outcomes: Practical Guidance for Cox Proportional Hazards Models. Ann Intern Med.

[28] Chowdhury MZI, Turin TC (2020). Variable selection strategies and its importance in clinical prediction modelling. Fam Med Community Health.

[29] Harrell FEJ (2015). Regression Modeling Strategies.

[30] Steyerberg EW, Harrell FE, Borsboom GJ, Eijkemans MJ, Vergouwe Y, Habbema JD (2001). Internal validation of predictive models: efficiency of some procedures for logistic regression analysis. J Clin Epidemiol.

[31] Wiseman JT, Sarna A, Wills CE, Beane J, Grignol V, Ejaz A (2022). Patient Perspectives on Defining Textbook Outcomes Following Major Abdominal Surgery. J Gastrointest Surg.

[32] van der Werf LR, Wijnhoven BPL, Fransen LFC, van Sandick JW, Nieuwenhuijzen GAP, Busweiler LAD (2019). A National Cohort Study Evaluating the Association Between Short-term Outcomes and Long-term Survival After Esophageal and Gastric Cancer Surgery. Ann Surg.

[33] van der Kaaij RT, de Rooij MV, van Coevorden F, Voncken FEM, Snaebjornsson P, Boot H (2018). Using textbook outcome as a measure of quality of care in oesophagogastric cancer surgery. Br J Surg.

[34] Sedlak K, Rawicz-Pruszynski K, Mlak R, Geca K, Skorzewska M, Pelc Z (2022). Union is strength: Textbook outcome with perioperative chemotherapy compliance decreases the risk of death in advanced gastric cancer patients. Eur J Surg Oncol.

[35] Bolger JC, Al Azzawi M, Whooley J, Bolger EM, Trench L, Allen J (2021). Surgery by a minimally invasive approach is associated with improved textbook outcomes in oesophageal and gastric cancer. Eur J Surg Oncol.

[36] Priego P, Cuadrado M, Ballestero A, Galindo J, Lobo E (2019). Comparison of Laparoscopic Versus Open Gastrectomy for Treatment of Gastric Cancer: Analysis of a Textbook Outcome. J Laparoendosc Adv Surg Tech A.

[37] Levy J, Gupta V, Amirazodi E, Allen-Ayodabo C, Jivraj N, Jeong Y (2020). Gastrectomy case volume and textbook outcome: an analysis of the Population Registry of Esophageal and Stomach Tumours of Ontario (PRESTO). Gastric Cancer.

[38] Mehta R, Tsilimigras DI, Paredes AZ, Sahara K, Moro A, Farooq A (2020). Comparing textbook outcomes among patients undergoing surgery for cancer at U. S. News & World Report ranked hospitals. J Surg Oncol.

[39] Hyer JM, Beane JD, Spolverato G, Tsilimigras DI, Diaz A, Paro A (2022). Trends in Textbook Outcomes over Time: Are Optimal Outcomes Following Complex Gastrointestinal Surgery for Cancer Increasing?. J Gastrointest Surg.

