# Supplementary material for: Evaluation of optimum classification measures used to define textbook outcome among patients undergoing curative-intent resection of gastric cancer
Source: BMC Cancer. 2023 Dec 6;23:1199. doi: 10.1186/s12885-023-11695-4 (PMC10701946; doi:10.1186/s12885-023-11695-4)
Supplement: Supplementary file 1 — Additional file 1: Table S1. Components used to define Textbook Outcome (TO) in gastric cancer surgery. Table S2. Performance parameters of Cox proportional hazards models for Textbook Outcomes (TO) defined using various components (Kraków cohort, N = 1,479). Table S3. Cox proportional analysis for overall and conditional survival in patients with individual components of textbook outcome (Kraków cohort, N = 1,479). Table S4. Univariate analysis of overall and conditional survival in patients with individual components of textbook outcome (TO) (Kraków cohort, N = 1,479). Table S5. Univariate and multivariate Cox proportional hazards analysis for overall survival (Kraków cohort, N = 1,479). Figure S1. Study flowchart. Figure S2. Kaplan–Meier survival curves for overall survival of patients achieving each quality metric of Textbook Outcome for Kraków cohort, N = 1,479 (log-rank test). Figure S3. Kaplan–Meier survival curves for conditional survival of patients achieving each quality metric of Textbook Outcome for Kraków cohort, N = 1,479 (log-rank test). Figure S4. LOESS curve fitting (solid line with 95% confidence intervals) for the temporal trend in the annual proportion of patients achieving each quality metric of Textbook Outcome (Kraków cohort, N = 1,479). Figure S5. Distribution of postoperative complications according to the Clavien-Dindo classification in different time periods (Kraków cohort, N = 1,479). Figure S6. Distribution of postoperative complications according to the Clavien-Dindo classification by the type of resection (Kraków cohort, N = 1,479). [file 12885_2023_11695_MOESM1_ESM.docx]

Table S1.

Components used to define Textbook Outcome (TO) in gastric cancer surgery

| **TO component** | **Author, year** | | | | | |
| --- | --- | --- | --- | --- | --- | --- |
|  | Busweiler 2017 | Levy 2022 | Roh 2021 | DalCero 2022 | Aquina 2021 | Spolverato 2022 |
| Macroscopically complete resection according to the surgeon | + | ⎯ | ⎯ | + | ⎯ | ⎯ |
| No intraoperative complication | + | ⎯ | + | ⎯ | ⎯ | ⎯ |
| Microscopically radical (R0) resection | + | + | + | + | + | + |
| At least 15 lymph nodes retrieved and examined | + | + | + | + | + | + (≥16) |
| No severe postoperative complication (Clavien–Dindo grade II or higher) | + | + | + | + | ⎯ | ⎯ |
| No reintervention (surgical, endoscopic or radiological)  ≤ 30 days after surgery | + | + | + | + | ⎯ | ⎯ |
| No readmission to ICU  ≤ 30 days after surgery | + | + | + | ⎯ | ⎯ | ⎯ |
| Hospital stay ≤ 21 days | + | + | + | + (≤14 days) | + (19 days) | + (75^th^ percentile) |
| No postoperative mortality  ≤ 30 days after surgery | + | + | + | +(90 days) | ⎯ | + |
| No hospital readmission  ≤ 30 days after discharge | + | + | + | + | ⎯ | ⎯ |
| Chemotherapy compliance | ⎯ | ⎯ | ⎯ | ⎯ | +  (T3 or N+) | + (T3 or N+) |

Table S2. Performance parameters of Cox proportional hazards models for Textbook Outcomes (TO) defined using various components (Kraków cohort, N = 1,479)

| **TO Definition** | **No. of model components** | **AIC** | **BIC** | **c-index** |
| --- | --- | --- | --- | --- |
| Busweiler et al., 2017 | 10 | 13617.01 | 13661.73 | 0.7134 |
| Levy et al., 2022 | 8 | 13633.77 | 13668.54 | 0.7088 |
| DalCero et al., 2022 | 8 | 13675.61 | 13710.38 | 0.6984 |
| Aquina et al., 2021 | 4 | 13786.45 | 13801.35 | 0.6304 |
| Roh et al., 2021 | 9 | 13632.53 | 13672.27 | 0.7101 |
| Spolverato et al., 2022 | 4 | 13748.97 | 13763.88 | 0.6734 |

Table S3. Cox proportional analysis for overall and conditional survival in patients with individual components of textbook outcome (Kraków cohort, N = 1,479)

| **Parameter** | **Overall survival** | | **Conditional survival^*^** | |
| --- | --- | --- | --- | --- |
|  | HR (95%CI) | *P* | HR (95%CI) | *P* |
| **Resection with curative intent** | 0.60 (0.47 – 0.77) | 0.001 | 0.60 (0.47 – 0.76) | 0.001 |
| No intraoperative complication | 0.68 (0.52 – 0.89) | 0.005 | 0.70 (0.54 – 0.91) | 0.008 |
| A microscopically radical (R0) resection | 0.50 (0.39 – 0.63) | 0.001 | 0.53 (0.42 – 0.66) | 0.001 |
| At least 15 lymph nodes retrieved | 0.88 (0.75 – 1.03) | 0.104 | 1.03 (0.89 – 1.20) | 0.664 |
| No severe postoperative complication | 0.68 (0.58 – 0.78) | 0.001 | 0.67 (0.58 – 0.78) | 0.001 |
| No reintervention | 0.62 (0.47 – 0.82) | 0.001 | 1.55 (1.17 – 2.04) | 0.002 |
| No readmission to ICU | 0.33 (0.24 – 0.45) | 0.001 | 0.22 (0.17 – 0.30) | 0.001 |
| Hospital stay ≤ 21 days | 0.57 (0.47 – 0.70) | 0.001 | 0.64 (0.53 – 0.77) | 0.001 |
| No hospital readmission ≤ 30 days | 0.96 (0.70 –1.32) | 0.818 | 0.99 (0.72 – 1.35) | 0.930 |

^*^excluding 30-day mortality

Table S4. Univariate analysis of overall and conditional survival in patients with individual components of textbook outcome (TO) (Kraków cohort, N = 1,479)

| **Number of achieved TO measures** | **Overall survival** | | **Conditional survival^*^** | |
| --- | --- | --- | --- | --- |
|  | HR (95%CI) | *P* | HR (95%CI) | *P* |
| 5 or less | reference |  | reference |  |
| 6 | 0.24 (0.18 – 0.31) | <0.001 | 0.33 (0.24 – 0.45) | <0.001 |
| 7 | 0.14 (0.11 – 0.18) | <0.001 | 0.20 (0.15 – 0.27) | <0.001 |
| 8 | 0.11 (0.09 – 0.14) | <0.001 | 0.16 (0.12 – 0.22) | <0.001 |
| 9 | 0.09 (0.07 – 0.11) | <0.001 | 0.13 (0.10 – 0.17) | <0.001 |
| 10 | 0.07 (0.05 – 0.08) | <0.001 | 0.10 (0.07 – 0.13) | <0.001 |

^*^excluding 30-day mortality

Table S5. Univariate and multivariate Cox proportional hazards analysis for overall survival (Kraków cohort, N = 1,479)

| **Parameter** | **Univariate analysis** | | **Multivariate analysis** | |
| --- | --- | --- | --- | --- |
|  | HR (95%CI) | *P* | HR (95%CI) | *P* |
| Age, >65 yrs | 1.67 (1.48 – 1.89) | <0.001 | 1.47 (1.29 – 1.68) | <0.001 |
| Sex, male | 1.27 (1.11 – 1.45) | <0.001 | 1.24 (1.08 – 1.43) | 0.002 |
| Weight loss, yes | 1.53 (1.35 – 1.74) | <0.001 | 1.16 (1.02 – 1.33) | 0.028 |
| ASA Class, 3 or 4 | 1.57 (1.36 – 1.80) | <0.001 | 1.41 (1.22 – 1.64) | <0.001 |
| Neoadjuvant chemotherapy, yes | 1.21 (0.99 – 1.50) | 0.067 | 1.07 (0.86 – 1.33) | 0.525 |
| Gastrectomy, yes | 1.42 (1.25 – 1.61) | <0.001 | 0.88 (0.75 – 1.03) | 0.120 |
| D2 lymphadenectomy, yes | 0.83 (0.72 – 0.94) | 0.005 | 0.82 (0.70 – 0.95) | 0.008 |
| Surgeon caseload, >100 | 0.97 (0.83 – 1.12) | 0.638 | 0.86 (0.74 – 1.00) | 0.055 |
| Adjacent organ resection, yes | 1.74 (1.53 – 1.97) | <0.001 | 1.15 (0.98 – 1.35) | 0.080 |
| RBC transfusion, yes | 1.72 (1.52 – 1.95) | <0.001 | 1.04 (0.90 – 1.21) | 0.585 |
| Tumour location, distal | 0.79 (0.69 – 0.89) | <0.001 | 0.95 (0.82 – 1.10) | 0.490 |
| Tumour size, >70 mm | 2.11 (1.87 – 2.39) | <0.001 | 1.10 (0.95 – 1.27) | 0.200 |
| Histological type, intestinal | 0.80 (0.71 – 0.90) | <0.001 | 0.86 (0.75 – 0.98) | 0.021 |
| Tumour grade, 2 or 3 | 1.62 (1.34 – 1.96) | <0.001 | 0.96 (0.77 – 1.20) | 0.698 |
| Lymfovascular invasion, yes | 1.77 (1.56 – 2.00) | <0.001 | 1.17 (1.01 – 1.35) | 0.036 |
| Perineural invasion, yes | 1.43 (1.22 – 1.68) | <0.001 | 1.05 (0.88 – 1.25) | 0.616 |
| Resection margins, positive | 3.11 (2.68 – 3.62) | <0.001 | 1.39 (1.15 – 1.69) | <0.001 |
| pT category (AJCC)  T1a  T1b  T2  T3  T4a  T4b | —  0.93 (0.64 – 1.35)  1.31 (0.93 – 1.85)  2.52 (1.90 – 3.35)  3.88 (2.86 – 5.26)  5.56 (4.07 – 7.59) | 0.706  0.128  <0.001  <0.001  <0.001 | —  0.90 (0.61 – 1.32)  1.15 (0.79 – 1.67)  1.70 (1.21 – 2.38)  1.75 (1.21 – 2.54)  2.03 (1.37 – 2.99) | 0.595  0.455  0.002  0.003  <0.001 |
| pN category (AJCC)  N0  N1  N2  N3a  N3b | —  1.66 (1.35 – 2.05)  2.15 (1.76 – 2.63)  3.00 (2.53 – 3.57)  4.69 (3.93 – 5.61) | <0.001  <0.001  <0.001  <0.001 | —  1.20 (0.96 – 1.51)  1.77 (1.42 – 2.21)  2.19 (1.78 – 2.69)  2.66 (2.13 – 3.33) | 0.110  <0.001  <0.001  <0.001 |
| Textbook outcome, achieved | 0.53 (0.46 – 0.61) | <0.001 | 0.61 (0.51 – 0.73) | <0.001 |

Figure S1. Study flowchart.

Figure S2. Kaplan–Meier survival curves for overall survival of patients achieving each quality metric of Textbook Outcome for Kraków cohort, N = 1,479 (log-rank test).

Figure S3. Kaplan–Meier survival curves for conditional survival of patients achieving each quality metric of Textbook Outcome for Kraków cohort, N = 1,479 (log-rank test).

Figure S4. LOESS curve fitting (solid line with 95% confidence intervals) for the temporal trend in the annual proportion of patients achieving each quality metric of Textbook Outcome (Kraków cohort, N = 1,479).

Figure S5. Distribution of postoperative complications according to the Clavien-Dindo classification in different time periods (Kraków cohort, N = 1,479).

Figure S6. Distribution of postoperative complications according to the Clavien-Dindo classification by the type of resection (Kraków cohort, N = 1,479).
